# Palliative care development as a solution to mitigate the human crisis of cancer in low- and middle-income countries: a theory of change approach

**DOI:** 10.1007/s00520-026-11306-5

**Published:** 2026-10-09

**Authors:** Seema Rajesh Rao, Shrikant Atreya, Mebin Mathew, Arathi P. Rao, Catherine Walshe, Nancy Preston, Srinagesh Simha, Richard Sullivan, William E. Rosa, Gary Rodin, Naveen Salins

**Affiliations:** 1 Manipal Hospice and Respite Centre, Manipal Academy of Higher Education, Manipal, India; 2 Department of Palliative Care and Psycho-Oncology, Tata Medical Centre, Kolkata, India; 3 Department of Palliative Medicine and Supportive Care, Kasturba Medical College, Manipal Academy of Higher Education, Manipal, India; 4 Department of Global Public Health Policy and Governance, Prasanna School of Public Health, Manipal Academy of Higher Education, Manipal, India; 5 Department of Health Research, Lancaster University, Lancaster, UK; 6 Bangalore Hospital Trust – Karunashraya, Bangalore, Karnataka India; 7 Institute of Cancer Policy, Centre for Conflict & Health Research, King’s College London, London, UK; 8 Department of Psychiatry and Behavioral Sciences, Memorial Sloan Kettering Cancer Centre, New York City, NY USA; 9 Division of Consultation Liaison Psychiatry, Princess Margaret Cancer Centre, University Health Network, University of Toronto, Toronto, Ontario Canada

**Keywords:** Palliative care development, Lower-middle-income countries, Systems transformation, Theory of change, Health system integration

## Abstract

**Background:**

The burden of cancer is rising in low- and middle-income countries (LMICs), where social determinants, limited access to treatment, and inadequate palliative care amplify suffering. With most unmet palliative care needs concentrated in LMICs, strengthening palliative care in these settings is a moral and health systems imperative. This study aimed to develop a roadmap for palliative care development in LMICs.

**Methods:**

A theory of change framework was used to explore palliative care development in LMICs as it enabled mapping of complex, interacting elements and their impact on meaningful change. Stakeholders were recruited through purposive and snowball sampling via the research team’s professional networks, and included physicians, nurses, public health experts, and community participants. Eight virtual workshops addressed key elements of the theory of change framework: impact, outcomes, preconditions, interventions, and assumptions. Data from workshop transcripts, online chats, and facilitator meetings were analysed iteratively using consensus-driven theory-informed approach to map the causal pathway.

**Results:**

Twenty-one stakeholders from three continents participated in eight facilitated workshops. Stakeholders emphasised integration of palliative care into health systems as central to its development and essential for mitigating cancer-related suffering. The desired outcome was comprehensive, sustainable, and culturally relevant palliative care. Fourteen preconditions, 21 interventions, and 12 assumptions were identified and mapped onto the causal pathway.

**Conclusion:**

The theory of change approach facilitated the creation of contextually grounded stakeholder-informed roadmap for palliative care development in LMICs, achievable through integration within existing health systems. Future efforts should concentrate on testing the implementation, feasibility and impact of this roadmap across diverse LMICs.

## Introduction

The burden of cancer is rising globally [1], with approximately 20 million new cases and 9.7 million deaths in 2022 [2]. While high-income countries (HICs) report more cases, cancer-related mortality has either decreased or stabilised in these regions [3]. However, low- and middle-income countries (LMICs) face a dual challenge, of rising cancer burden and cancer-related mortality, with 75% of all cancer deaths projected to occur in LMICs by 2030 [4, 5]. This disparity reflects the systemic inequities inherent within LMICs, such as limited health literacy, poor risk factor control, inadequate screening and delayed presentation, and poor survival due to inaccessible and unaffordable cancer care [6].

The Lancet Commission on Palliative Care and Pain Relief estimates that over 90% of patients dying from cancer experience serious health-related suffering (SHS), with the need for palliative care growing rapidly in LMICs [7, 8]. Social determinants of health such as race, gender, ethnicity, culture, socioeconomic status, geographic location, housing, food and security, and accessibility and affordability of healthcare can impact cancer outcomes and experience of suffering [9]. Various studies have shown that patients and families in resource-constrained settings experience increased physical and psychological symptom burden, financial toxicity, caregiver burden, and reduced workforce participation [10]. Catastrophic out-of-pocket expenditures associated with cancer care exacerbate distress and deepen inequities [11]. Consequently, diagnosis of cancer in LMICs represents a biomedical, social, and an economic crisis [12].

The global burden of SHS is projected to double by the year 2060 [10]. Palliative care and pain relief are effective in partially alleviating SHS and improving the quality of life (QoL) for patients and their families [7]. High-income countries have shown improved palliative care development and integration in accordance with oncology guidelines and other expert recommendations [13–15]. It is estimated that SHS in these countries will likely slow or even plateau by 2050 [10]. However, this is lacking in many LMICs, where the end of life is marked by unrelieved pain and suffering [16]. Policy gaps, clinical, organisational, and system barriers including an inadequately trained workforce, lack of funding, inadequate access to medications, and lack of both provider and public awareness of palliative care, along with stigma about cancer and death and dying, impede palliative care development and integration in these countries [17]. Therefore, solutions for palliative care development within LMICs are likely to be multifaceted and complex, with multiple, discrete, interrelated components [18].

By actively engaging potential stakeholders involved in transforming themselves and their environment (those involved in palliative care development in their countries) and by identifying the innate resources and mechanisms that drive change within systems, we hope to develop a palliative care model, that is pragmatic and responsive to LMIC context, with the potential to mitigate SHS caused by cancer [19, 20]. The aim of this study is to identify the drivers of the imbalance and gaps in palliative care development in LMICs and to develop a causal pathway for an ideal palliative care model for low-resource settings through a theory of change approach. This work was undertaken as part of the Lancet Oncology Commission on the human crisis of cancer and is aligned with its mandate of addressing global inequities in cancer care by strengthening the development and integration of palliative care within oncology in LMICs [21].

## Methods

### Design

The theory of change framework is used to explore palliative care development in the LMICs, as it enables systematic mapping of the complex interacting elements and illustrates ‘how’ and ‘why’ a desired change (i.e. palliative care development and integration) is expected to happen in a given context (LMICs) [22]. It employs a backward mapping approach beginning with the desired impact or goal, followed through with long-term outcomes, preconditions (i.e. short-term or intermediate outcomes), interventions needed to achieve these outcomes, and facilitators and barriers to implementation [22]. The common terminologies used in the theory of change map are presented in Table 1 [22, 23]. This approach is used to generate solutions for complex social problems and to develop hypothetical causal pathways that show connections between interventions and their anticipated outcomes [24]. Theory of change in the present study uses a rigorous, multi-stakeholder, participatory approach, requiring stakeholders to set clear long-term goals, formulate actions to achieve these goals, and identify preconditions, measurable indicators of success, assumptions, and rationales, while arranging these in a causal framework diagram [22]. The Aspen Institutes and De Silva et al.’s practical guides were followed to develop the theory of change model for this study [25].

**Table 1:** Common terminologies in theory of change

Terminology	Definition
Impact (goal)	Ultimate outcome or the real-world change that the program is trying to achieve
Long-term outcome	Outcome that the program can achieve on its own
Preconditions	Intermediate outcomes, precursors, or requirements that need to be fulfilled to achieve the desired outcome
Interventions	‘Action plan’ that the program must undertake to bring about the preconditions (outcomes). Forms the different components of complex interventions
Indicators	Outcome measures that are determined and documented for each outcome. They help evaluate whether the program is making progress or achieving the intended outcome
Ceiling of accountability	Level beyond which indicators are no longer used to measure achievement of outcomes. Program stops accepting responsibility for achieving the outcomes. Drawn between impact and long-term outcomes in the theory of change map
Assumptions	External conditions beyond the control of the program that must exist for outcomes to be achieved

### Setting

This research was conducted across LMICs globally through a series of virtual workshops held over 3 months, involving stakeholders across Asia, Africa, and South America. Stakeholders were drawn from diverse healthcare settings, including hospital and community-based, reflecting varied resource constraints and service delivery environments of LMICs. For the purpose of this study, LMICs were defined using the World Bank Atlas income classification applicable at the time of recruitment.

### Population

Stakeholders included individuals with direct experience in palliative care development or delivery in LMICs and included both generalist and specialist providers. The inclusion and exclusion criteria are specified in Table 2.

**Table 2:** Inclusion and exclusion criteria

Inclusion criteria	Exclusion criteria
Aged 18+, no maximum age AND	Palliative care providers working exclusively in a non-cancer setting
Based within a low- and middle-income country according to the World Bank^1^ AND	Family caregivers with experience in caregiving for a person with non-cancer condition
Involved in generalist and/or specialist palliative care for those with advanced cancer as a provider (health or social care professional) policymaker, funder or manager of care OR in receipt of care as a patient and/or family member of a patient with advanced cancer AND	
Sufficient working knowledge of English to be able to contribute to a workshop	

^1^For the current 2024 fiscal year, low-income economies are defined as those with a GNI per capita, calculated using the World Bank Atlas method, of $1135 or less in 2022; lower middle-income economies are those with a GNI per capita between $1136 and $4465 and listed online here:  https://datatopics.worldbank.org/world-development-indicators/the-world-by-income-and-region.html

### Sampling and recruitment

To ensure diverse participation, an initial group of stakeholders were purposively recruited through the research team’s professional networks which included collaborators, academic partners, and palliative care practitioners in LMICs. Recruitment was not country-specific, but country representation emerged organically through purposive and subsequent snowball sampling. Participants were identified a priori based on their demonstrable involvement in palliative care development in their region. They were either involved in either establishing palliative care or scaling up services, implementing or managing programs, education and training, policy development and advocacy, or palliative care research. Additional stakeholders were recruited through snowball sampling, with the initial stakeholders recommending other participants.

All stakeholders were invited via emails and phone calls and were provided information about the workshop process and expectations. Those who were accessible, available, and willing to participate were recruited. Consent was obtained from all participants and facilitators for recording the workshops.

### Data collection

The workshops were facilitated by a team of eight experts: six palliative care researchers (NS, CW, NP, SRR, SA, SNS), one public health expert (AR), and one support staff (MM). The researchers (CW, NP, NS, SRR) from the UK and India, familiar with the theory of change framework, facilitated the workshops.

A facilitated workshop model was used, with facilitators and stakeholders co-creating content for a predefined deliverable [26]. Each workshop focused on a specific predefined component of the causal pathway and was led by one member of the research team [27]. The workshops were evidence-informed but stakeholder led. Across workshops, the facilitators presented relevant theory of change examples, findings from the framework synthesis, existing palliative care models, and interventions and assumptions to provide background and context to guide discussions.

Given that there were 21 stakeholders in different time zones, the stakeholders were divided into two groups (Group A and Group B). The first three workshops were conducted for both groups separately and were led by different facilitators. In the final two workshops, the groups were merged for logistical convenience. If stakeholders were absent, relevant input from them was collected via email. Multiple team meetings between facilitators were held between workshops. These meetings focused on reviewing the materials developed in the facilitated workshops and aligning it with predefined deliverables and the causal pathway.

A summary of the facilitated workshops is provided in Table 3.

**Table 3:** Summary of theory of change workshops

Items	Workshop 1 (× 2)	Workshop 2 (× 2)	Workshop 3 (× 2)	Workshop 4	Workshop 5	
Total number of Participants	21	21	19	16	14	
Central theme	Impact	Long-term outcome	Interventions	Assumptions	Theory of change map refinement	
Structure	Established rapport and introduced the theory of change framework	Recap and validation of the draft impact statement	Recap and agreement on the long-term outcomes	Recap and agreement on interventions	Presentation and consensus about the theory of change map	
	Defined the intended impact of palliative care in mitigating SHS in cancer in LMICs	Facilitators introduced existing models and long-term outcomes related to palliative care development	Facilitators presented existing interventions across settings	Facilitators presented presuppositions and assumptions impacting palliative care development		Stakeholders reviewed and refined the theory of change map
	Facilitated discussion to define the desired impact	Facilitated discussion to identify and refine the long-term outcomes	Stakeholders identified ‘action plans/interventions to achieve the desired outcomes	Stakeholders Identified assumptions influencing implementation		Final consensus on the causal pathways
Outcome	Agreement on the impact statement	Three long-term outcomes and their associated attributes were identified	Fourteen preconditions and 21 key interventions were identified	Twelve assumptions identified		Agreement on the theory of change map and causal pathways
				Contextual factors and the ceiling of accountability delineated		

#### Workshop 1: Rapport, introduction, and impact

The first workshop focused on establishing rapport and introducing the purpose, structure, and key components of the theory of change. A sample map about integrating palliative care in the ICU in an LMIC was used to demonstrate the application of the theory of change [28]. This was followed by a presentation by one of the facilitators on the overall findings of a framework synthesis on palliative care development in LMICs and its impact on the human crisis of cancer [29].

A facilitated discussion followed on the impact of palliative care in mitigating cancer-related SHS in LMICs. Stakeholders individually identified the real-world palliative care solutions for mitigating cancer-related suffering based on their experience in palliative care development and delivery while also considering the evidence presented in the framework synthesis. Similar workshops were conducted for both Group A and Group B. Post-workshop the stakeholder inputs were analysed. Although the focus was on impact, several preconditions, long-term outcomes, intervention activities, and assumptions emerged implicitly from the discussion. These ideas were grouped and categorised into the relevant components of the theory of change framework.

#### Workshop 2: Long-term outcomes

The second workshop started with a recap of the previous session and the impact statement developed by the research team based on stakeholder inputs from Workshop 1. A poll was conducted to assess the stakeholders’ agreement with the impact statement. All 16 stakeholders across both groups who participated in Workshop 2 agreed with the impact statement, highlighting the influence of socioeconomic, cultural and political factors on outcomes.

This workshop further focused on developing a consensus on long-term solutions for the palliative care development in LMICs. A facilitator presented existing long-term solutions, both globally and in LMICs for mitigating cancer-related suffering. This was followed by facilitated discussions. Stakeholder responses were recorded verbally and via chat across both groups.

#### Workshop 3: Interventions

The third workshop explored stakeholders’ perspectives on the actions required to achieve the long-term outcomes identified in Workshop 2, as well as the contextual factors influencing the outcomes. To provide a contextual framework, a facilitator presented various palliative care interventions in the context of cancer across various healthcare settings, including their composition, timing, point of initiation, setting, level, and type of care. Following this, the stakeholders were invited to discuss feasible and contextually relevant interventions that could achieve the proposed outcomes in LMICs. Similar workshops were conducted for both the groups.

#### Workshop 4: Assumptions/presuppositions and preconditions

The fourth workshop brought both the groups together and explored stakeholders’ views on the assumptions and preconditions that could either facilitate or impede the development of palliative care in LMICs. One of the facilitators presented the various presuppositions/assumptions about palliative care internationally. This was followed by a facilitated discussion about how these presuppositions (assumptions) and preconditions might facilitate or hinder palliative care development in the context of cancer care in LMICs. A preliminary theory of change map was presented to the stakeholders for feedback and refinement.

#### Workshop 5: Future planning

In the final workshop, facilitators summarised the prior discussions and outlined the key achievements. The completed theory of change map was presented to the 14 stakeholders present in the workshop. Input from others was sought via email. The stakeholders further reviewed and refined the theory of change map, offering input on each component of the causal pathway.

### Data analysis

All workshops were video recorded and transcribed verbatim. Workshop minutes, facilitators’ notes, chat box posts, email communications, and the contents of the post-workshop debrief were collected for data analysis. Data triangulation enabled comprehensive understanding of the complex concepts discussed, strengthening the credibility and robustness of the findings.

Data analysis occurred concurrently with the workshop process through multiple team meetings interspersed between the workshops. An iterative theory-driven analytical approach, consistent with established theory of change methodology, was used to synthesise stakeholder inputs [22]. The research team reviewed the data and identified emerging concepts. These concepts were categorised into theory of change components: preconditions, interventions, assumptions, and expected impacts through a consensus-driven approach. Relationships between these concepts were examined and systematically mapped into the causal pathway to illustrate the logical relationship between activities, outcomes, and impact. The stakeholders and facilitators further refined this evolving model iteratively. This was then mapped and graphically represented in the theory of change map.

### Ethics approval

Ethical approval was obtained from the Lancaster University Faculty of Health and Medicine Research Ethics Committee (FHM-2024-4402-RECR-2) on 3.5.2024 prior to the commencement of the workshops. Stakeholders provided informed consent for workshop participation, audio and video recordings. All data was handled confidentially.

## Results

Twenty-one stakeholders from 12 countries (India, Bangladesh, Sri Lanka, Nepal, Indonesia, Bhutan, Ghana, Rwanda, Malawi, Brazil, Mexico, and Colombia), participated in eight workshops over 3 months. Each workshop lasted over 2 h. The diversity of the stakeholders enabled incorporation of varied perspectives, experiences and expertise across healthcare systems and culture. Four facilitators who led the workshop had prior knowledge about the theory of change framework.

The details of the stakeholders are provided in Table 4.

**Table 4:** Characteristics of the study participants in the theory of change workshops

Characteristics		Number of participants *N* = 21
**Gender**		
Male		12
Female		9
**Country**	**Stage of palliative care development**^1^	
Bangladesh	Progressing	2
Sri Lanka	Progressing	1
Nepal	Emerging	2
Indonesia	Established	2
Ghana	Progressing	1
Colombia	Established	2
Rwanda	Established	2
Bhutan	Progressing	1
Brazil	Established	1
Mexico	Established	1
India	Progressing	5
Malawi	Established	1
**Profession**		
Palliative care physician		11
Oncologist		1
Public health expert		1
Nurse/researcher		3
Community participants		1
Family medicine physician		3
Paediatrician		1

^1^Tripodoro VA, Fidalgo JFL, Pons JJ, Connor SR, Garralda E, Bastos F, et al. First-Ever Global Ranking of Palliative Care: 2025 World Map Under the New WHO Framework. J Pain Symptom Manage. 2025 Nov 1;70[5]:447–58. https://doi.org/10.1016/j.jpainsymman.2025.07.026

**Fig. 1:**
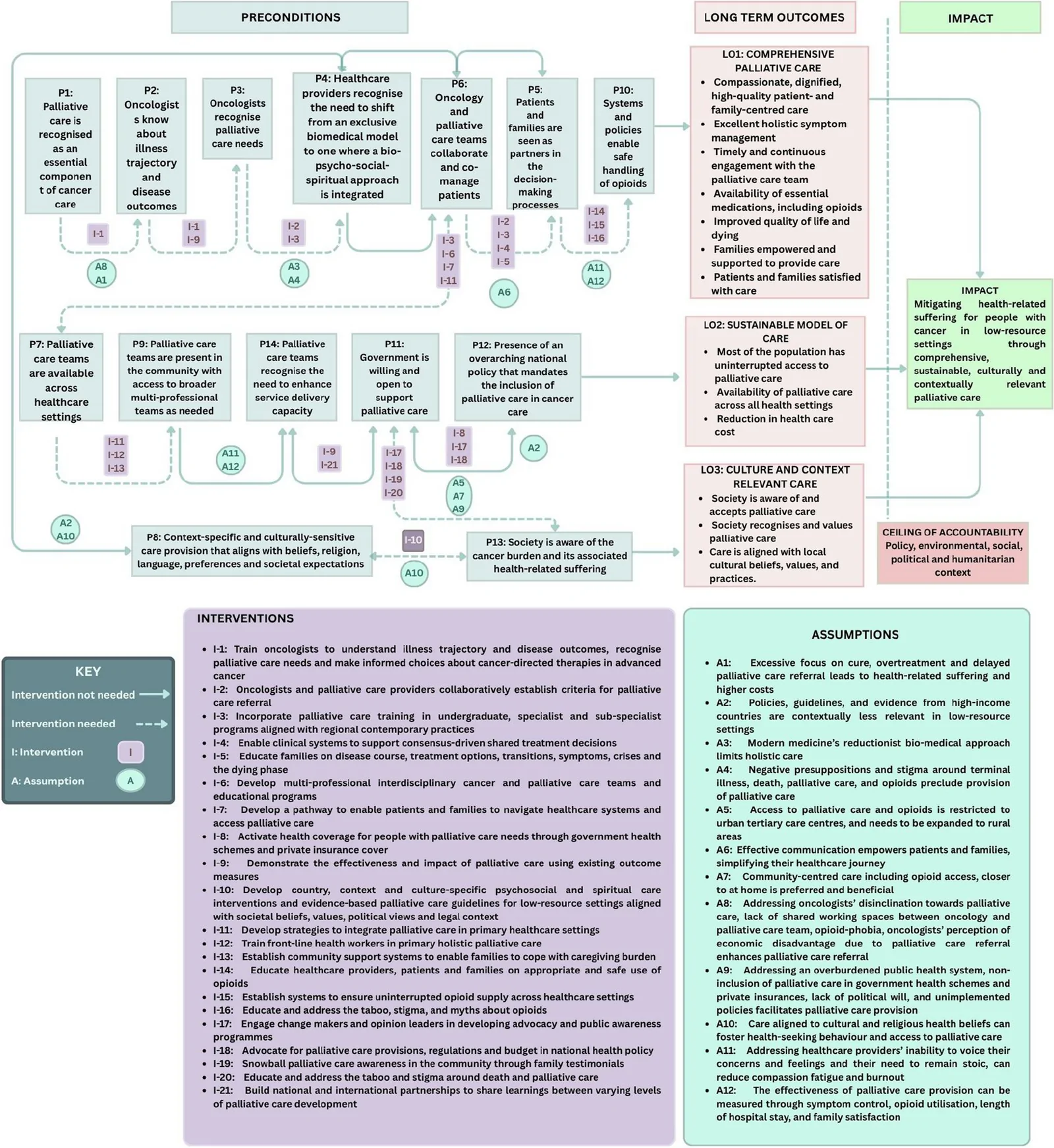
The theory of change map for palliative care development to alleviate health-related suffering in the context of cancer. Note: Interventions and assumptions may influence more than one precondition; the figure displays the principal linkage(s) for readability

A structured framework for reporting theory of change is provided by the Checklist for Reporting Theory of Change, which encompasses the following: (a) impact, (b) long-term outcomes, (c) preconditions, (d) ceiling of accountability, (e) interventions, and (f) assumptions [30]. The final approved theory of change map is presented in Fig. 1.

During the first workshop, the stakeholders identified the development of comprehensive, sustainable, and culturally relevant palliative care in LMICs as the desired impact required to mitigate SHS caused by cancer. Three key long-term outcomes were identified in the impact statement, and the attributes of each outcome, as explicated from the subsequent workshop transcripts, are outlined in Table 5.

**Table 5:** Long-term outcomes identified in the theory of change workshops

Domain	Core focus	Key components/indicators
Comprehensiveness	Patient- and family-centred, holistic care across the illness trajectory	• Compassionate, dignified, and high-quality care • Holistic symptom management (physical, psychological, social, spiritual) • Timely referral and sustained collaboration between oncology and palliative care teams • Reliable access to essential medications, including opioids • Empowerment and structured support for families in caregiving roles • Improved quality of death and dying • High levels of patient and family satisfaction with care
Sustainability	Continuity, affordability, and system-level integration	• Uninterrupted access to palliative care services across settings (hospital, home, hospice) • Affordable care models for most of the population • Consistent availability of essential palliative medicines, including opioids
Cultural & Contextual Relevance	Social acceptance and meaningful uptake of care	• Care aligned with local cultural beliefs, values, and practices • Sensitivity to family structures, decision-making norms, and caregiving patterns • Enhanced societal acceptance and valuation of palliative care

In the second and third workshops, the stakeholders identified integration of palliative care within existing healthcare systems in LMICs as the key mechanism that could lead to comprehensive, sustainable, and culturally relevant palliative care development. They also identified the 14 preconditions, 21 interventions, 12 assumptions, and the contextual features that could either enable or impede this integration, which were represented in the theory of change map.

The theory of change map focuses on the need to build strong systems (structure) and effective actions (processes) to develop comprehensive and sustainable palliative care. The stakeholders highlighted that evidence from high-income countries is not directly relevant to LMICs, due to disparities in patient demographics, healthcare delivery models, and societal values. The ceiling of accountability represents the threshold beyond which interventions are no longer directly responsible for achieving the desired impact [22]. In this study, the ceiling of accountability was situated between the long-term outcomes and the impact. External influences, including national policies, environmental conditions, and socio-political contexts including armed conflict, forced displacement, humanitarian emergencies, natural disasters, and major economic shocks, significantly impact the development and delivery of palliative care in LMICs. 

## Discussion

A systems thinking lens was used to interpret the study findings [31]. Systems thinking examines how various components of a system interact to generate patterns of behaviour and results [31]. When viewed through this perspective, the development and integration of palliative care in LMICs are a complex and non-linear intervention, that depends on a complicated network of interconnected factors. Health beliefs, socioeconomic conditions, healthcare delivery structures, workforce capacity, access to medications, and healthcare policies collectively shape SHS in cancer [17]. The theory of change workshops functioned as a systems-mapping exercise, enabling stakeholders to explicate these variables, understand the pattern of relationships among them, and identify the causal pathways, feedback loops, and path dependencies that contribute to these patterns. Importantly, culture emerged as an embedded determinant which influenced system behaviour across all levels. Our findings provide a context-specific articulation of how palliative care development unfolds within LMICs. The co-created systems map moves beyond generic descriptions to provide a more granular and operational understanding of palliative care development and integration in LMICs.

The theory of change workshops identified palliative care development and integration in LMICs as the key driver for mitigating serious health-related suffering in cancer. Evidence from high-income countries has shown that early and systematic integration of palliative care within oncology was beneficial and achievable [32–34]. However, persistent gaps in policy, knowledge, and implementation have impeded this integration in LMICs [17]. Viewed from a systems perspective, these gaps are not isolated phenomena, but the result of suboptimal system configuration, where structural components and relational dynamics interact to produce fragmented care. For example, limited opioid access is not an isolated phenomenon, but rather operates within a reinforcing feedback loop. Restrictive opioid policies fuel opiophobia among healthcare providers and caregivers, leading to reduced prescription and consumption of opioids. With fewer people using opioids, the issue of access loses prominence in political and healthcare dialogues, resulting in limited impetus for systems to change behaviour. This creates a self-reinforcing feedback loop in which restrictive policies continue to sustain and limit opioid access and use over time [35]. This highlights how systems behaviour emerges from relational dynamics among variables rather than from individual components [31]. Studies have shown that these systems behaviours remain deeply entrenched, limiting the adoption and implementation of new policies [7]. To bring about meaningful systems change, we need to identify and reconfigure the feedback loops that shape behaviour rather than trying to change isolated components.

Comprehensive care in the context of this workshop was defined as multidimensional, patient- and family-centred holistic care, enabled by timely referrals and seamless collaboration among oncology, palliative, and primary care teams [36]. Given the limited availability of specialist palliative care teams in LMICs, a tiered model of care may be required, with core palliative care competencies embedded within primary care, oncology, and other frontline services, with specialist palliative care team providing support for complex needs and systems leadership. Comprehensiveness also included equitable access to essential medications, including opioids, caregiver empowerment and support, improved quality of death and dying, and family satisfaction [36]. Comprehensive care was not feasible in environments where affordability, accessibility, and awareness were fragmented or uneven [34]. From a systems perspective, comprehensiveness represents an emergent property of coordinated subsystems, that is, larger things emerging from smaller parts. Comprehensiveness requires systemic coherence, where policies, processes, and practices are structurally and functionally aligned, and not isolated activities [37]. Studies on integrated care have consistently shown that for care to be comprehensive along with clinical coordination, alignment among funding mechanisms, workforce capacity, referral systems, and supply chains is essential.

Sustainability was identified as the foundational backbone of comprehensive palliative care. A sustainable and resilient healthcare ecosystem is equitable, affordable, and adaptable across all levels of the system: micro, meso, and macro [38]. In the theory of change workshops, stakeholders identified robust national policies, resource and budget allocation, effective governance, and political and legal commitment as determinants that enabled palliative care integration. Studies have shown that without these structural determinants at the macro-level, any intervention at the micro- or meso-level remain vulnerable to discontinuity [39]. At the meso-level, organisational readiness was achieved through workforce capacity building, infrastructure development, access to essential medications and opioids, and interprofessional collaborations among specialist oncology, palliative care, and primary care teams.

Systems theory highlights the crucial need to manage boundaries between oncology, palliative and primary care teams in order to reduce fragmentation of care [40, 41]. Interventions that strengthen coupling among subsystems improve coordination of care, facilitate translation of policy into practice, and are consistent with implementation science findings [40, 42]. At the microlevel, person- and family-centred interventions, good communication, community-engagement, and training of healthcare providers improve awareness and acceptance of palliative care. These interventions shape both behaviours (how people seek and use palliative care) and relationships (how patients, families, and providers interact). Higher-level macro and meso systems impact care delivery at the micro level, while real-world care experiences at the micro level may in turn shape these systems. Thus, palliative care development and integration are a collective responsibility and vertical misalignment across system levels can lead to implementation drift and reform fatigue [37, 39, 43].

Culture and context have emerged as central elements that impact every aspect of the causal pathway. For palliative care to be acceptable in LMICs, it must resonate with the local culture and align with the health beliefs of the local population [7, 44]. Culture influences how individuals experience and cope with illness, caregiving, and dying, and how they find meaning in suffering [45, 46]. Decision-making, acceptance of death, expressions of grief, communication of prognosis, and responsibility of care are shaped by cultural beliefs, social norms, and community practices [44–46]. In many LMICs, collusion between healthcare providers and families, where patients are not informed about the diagnosis or prognosis, is a major barrier to advanced care planning and early palliative care referral [47, 48]. Opiophobia is prevalent among both healthcare providers and the public [7, 49]. Attitudes towards cancer and its treatment, and belief in alternative or traditional systems of medicine, influence health-seeking behaviour [7, 44, 50]. From systems perspective, culture emerged as an embedded normative determinant that influenced system dynamics at all levels. For palliative care interventions to be sustainable, they need to be informed by local values and customs, delivered in a culturally sensitive manner, championed by the local community, and structured to account for local resource constraints.

The World Health Organization (WHO) identified six essential components for palliative care development, namely, integration of palliative care into health services, health policy, education and training, availability of essential medications, research, and empowerment of people and communities [51]. The tentative roadmap for strengthening palliative care in LMICs generated through the theory of change workshops resonates closely with this framework. Our findings extend this model by demonstrating how these elements interact dynamically within complex health and social systems in LMICs. By interpreting our findings through a systems thinking approach and situating the components of the theory of change frameworks within feedback loops, culture, and multilevel system structures, we highlight the need for a coordinated system transformation to alleviate SHS in cancer. Our study therefore not only reaffirms the principles of the WHO model for palliative care but also provides a contextually grounded systems framework for translating these principles into practice in LMICs.

## Strengths and limitations

The theory of change workshops engaged stakeholders from multiple regions (Asia, South America, and Africa) and disciplines (physicians, nurses, community participants). The cross-sectoral, multidisciplinary, and multi-system representation brought in a broad range of perspectives improving the generalisability of our findings. The participatory and iterative study design helped stakeholders incorporate their real-world experience to co-create solutions. This resulted in solutions that were contextually informed, acceptable, adaptable, and actionable across diverse political systems, culture, and healthcare settings. The study thus provided a strategic and pragmatic roadmap for palliative care integration in LMICs.

One of the key limitations of the workshops was its online format. Stakeholder participation, engagement, and interaction are critical components of theory of change. Virtual format may have reduced the quality of the interaction. Inconsistent attendance may have disrupted the continuity and completeness of the discussions and led to fragmented inputs. South Asia was overrepresented in our study as compared to Eastern Europe and Pacific regions. Furthermore, LMICs are heterogenous with regard to health-system resources, palliative care development, culture, and disease burden. These can potentially impact the transferability of our findings across varied settings. In addition, as proficiency in English was an inclusion criterion, language-related selection bias may have occurred, potentially excluding other eligible participants and limiting the nuances with which some participants could express context-specific perspectives.

## Conclusion

The development of palliative care into healthcare systems in LMICs was considered essential for relieving the SHS caused by cancer. Theory of change workshops created a strategic roadmap for achieving this through integration within health systems. The systems thinking framework used to interpret the study findings reiterated that effective systems integration is only possible when we move beyond isolated programmatic interventions towards systems transformation, aligning systems across the micro, meso and macro-levels. Such alignment creates adaptive systems that are culturally sensitive, contextually relevant, grounded in local realities, supported by policies, and owned by the community. Future research needs to focus on operationalising this roadmap to evaluate the feasibility, acceptability and impact of its implementation in various healthcare settings in LMICs.

## Data Availability

The Study data can be made available by contacting the Corresponding author of this paper upon reasonable request.
